# Does acute inflammation triggered by infection promote cancer progression?

**DOI:** 10.1371/journal.pbio.3003962

**Published:** 2026-09-15

**Authors:** Felipe Valença-Pereira, Bryan Johnson, James DeGregori, Mercedes Rincon

**Affiliations:** 1 Department of Immunology and Microbiology, University of Colorado, Anschutz Medical Campus, Aurora, Colorado, United States of America; 2 Department of Biochemistry and Molecular Genetics, University of Colorado, Anschutz Medical Campus, Aurora, Colorado, United States of America

## Abstract

Chronic infections are associated with cancer incidence and progression in human epidemiological data, but less is known about how such infections might promote cancer progression. Recent data implicate acute infection with common respiratory viruses, such as influenza and SARS-CoV-2, in cancer progression, providing evidence that infection-driven inflammation can promote tumor expansion and dissemination in experimental systems. The resulting acute inflammatory cascade and dynamic immune cell reprogramming can rapidly remodel the tissue microenvironment to favor cancer cell survival, growth, and evasion of immune elimination. However, acute infections can also elicit anti-tumor immune responses. Defining how infections differentially skew immune programs to promote or restrain cancer progression will therefore be essential for developing future strategies to target this disease.

## Abstract 2

Acute infections have been recently linked to cancer progression in experimental systems, which is supported by epidemiological human data, but they can also elicit anti-tumor immune responses. This Unsolved Mystery highlights our gaps in understanding how infections differentially skew immune programs to promote or restrain cancer progression; addressing these gaps will provide new avenues for prevention and therapy.

## The intersection of infection and cancer

Our understanding of cancer progression has evolved in recent years. Cancer progression is not simply a stepwise accumulation of mutations, instead it is a complex interaction in which context matters, and the microenvironment is a key part of understanding cancer initiation, progression, and metastatic disease [1]. Infections or other insults that promote tissue damage and inflammation therefore have the potential to alter cancer progression at all stages.

The relationship between cancer and infectious diseases is complex. Some viral infections, such as the Epstein-Barr virus and human papillomavirus, can deliver oncogenes into infected cells, contributing to the eventual development of cancer [2]. Notably, rates of infection with these viruses far exceed those of their associated cancers, and factors such as other infections (e.g., malaria for Burkitt’s lymphoma [3] and venereal diseases for cervical cancers) highlight potential roles for other factors such as inflammation and immune suppression in cancer progression [4]. Indeed, other infections can cause long-term chronic inflammation that is thought to promote the development of cancers [5]. Although understanding how cancer-causing viruses and chronic infections might lead to cancer is of critical importance, in this Unsolved Mystery we focus on the interaction between acute infection and cancer progression.

The field has long been aware that acute infection can affect cancer progression, as demonstrated by case reports from the 1700s and 1800s describing the regression of patient tumors following an acute infection [6]: these were considered to be early forms of immune therapy. The most well-known efforts in early immune therapy include William Coley’s development of ‘Coley’s toxins’, which were injections of killed Gram-positive *Streptococcus pyogenes* and gram-negative *Serratia marcescens* [6]. While most early work on this subject suggested an anti-tumor response following acute infection, recent studies suggest that acute infectious diseases have the potential to promote tumor growth and cancer progression. These conflicting results reflect the mystery at the center of interactions between acute viral infection and cancer pathogenesis.

In addition to affecting cancer incidence, infections may also be able to promote the outgrowth of metastatic cells. Traditionally, metastasis has been thought of as the final step of cancer progression. However, increasing evidence has shown that primary tumors can seed secondary sites much earlier than previously thought [7]. These disseminated cancer cells (DCCs) can remain dormant for months to years before they resume proliferation and form overt metastatic disease [8]. DCCs seeded at secondary sites, whether as single cells or as small micrometastases, can remain largely quiescent (cellular dormancy). Metastatic lesions can also persist without progression (tumor mass dormancy), where cell proliferation is balanced equally by cell death, mediated by the immune system or programmed cell death [9]. Thus, cancer dormancy can be thought of as the persistence of residual asymptomatic cancer without progression despite the absence of therapy. Treatment of DCCs remains a significant challenge clinically, and even detecting DCCs in patients remains extremely difficult due to their low abundance.

Recent studies have begun to unravel both the mechanisms that maintain metastatic tumor cell dormancy, as well as the altered contexts and insults that can lead to their awakening. Inflammation caused by a variety of factors can induce the awakening of dormant DCCs [10–13]. In mouse models of dormant DCCs, tobacco smoke exposure, in part mediated by lipopolysaccharide (LPS)-induced inflammation, resulted in the awakening of DCCs mediated by the formation of neutrophil extracellular traps (NETs) [11]. Additional work on LPS-induced awakening of dormant DCCs suggests that cytokines secreted by cells of the adaptive immune system also have a role in promoting awakening [14]. Inflammation induced by chemotherapeutic agents such as cisplatin, doxorubicin, or bleomycin also promotes the awakening of DCCs [13,15]. Beyond these inflammatory cues, the structural environment of the metastatic niche also shapes DCC awakening [16,17]. Dormant cells often occupy a perivascular niche, where endothelial quiescence helps maintain dormancy and vascular remodeling and endothelial activation can provide pro-outgrowth signals that favor escape from dormancy [18–20]. In parallel, extracellular matrix (ECM) remodeling and fibroblast activation can stiffen and reorganize the metastatic niche, alter growth factor availability, and create a permissive stromal environment for metastatic reactivation [16,21,22]. Increased collagen abundance and crosslinking into fibrillar collagen have also been linked to dormant DCC activation and to an angiogenic switch that supports tumor outgrowth [22]. Notably, influenza virus infection has been associated with increased levels of collagen and angiogenic factors, suggesting that infection-driven and inflammation-driven mechanisms can perturb the structural integrity of the metastatic niche and might contribute to DCC reactivation [23].

Host responses to infections are complex, involving remodeling of and often a large expansion of both the innate and adaptive parts of the immune system, along with damage to and repair of infected tissue. Many decades of study have shown that cancers at all stages are strongly influenced by the immune system, ranging from tumor-promoting roles of inflammation to tumor elimination, such as through T-cell-mediated killing. Thus, there is little doubt that infections alter cancer evolution and pathogenesis. The question is in which direction and how. In this Unsolved Mystery, we discuss evidence linking infections to either cancer progression or regression, with a focus on acute infections. We also discuss the potential implications of these links. Can understanding how different acute infections promote cancer progression versus regression be leveraged to favor the latter? Can we, and should we, interfere with pathways induced during acute infections that promote cancer progression? Can cancer-cell-mediated suppression of anti-tumor immune responses during an acute infection be reversed, allowing the infection-mobilized adaptive immune response to mediate tumor regression instead of progression? These questions remain unanswered, and we hope that highlighting these gaps will encourage research towards answers.

## What evidence is there for a link between infections and cancer progression in humans?

**Fig 1:**
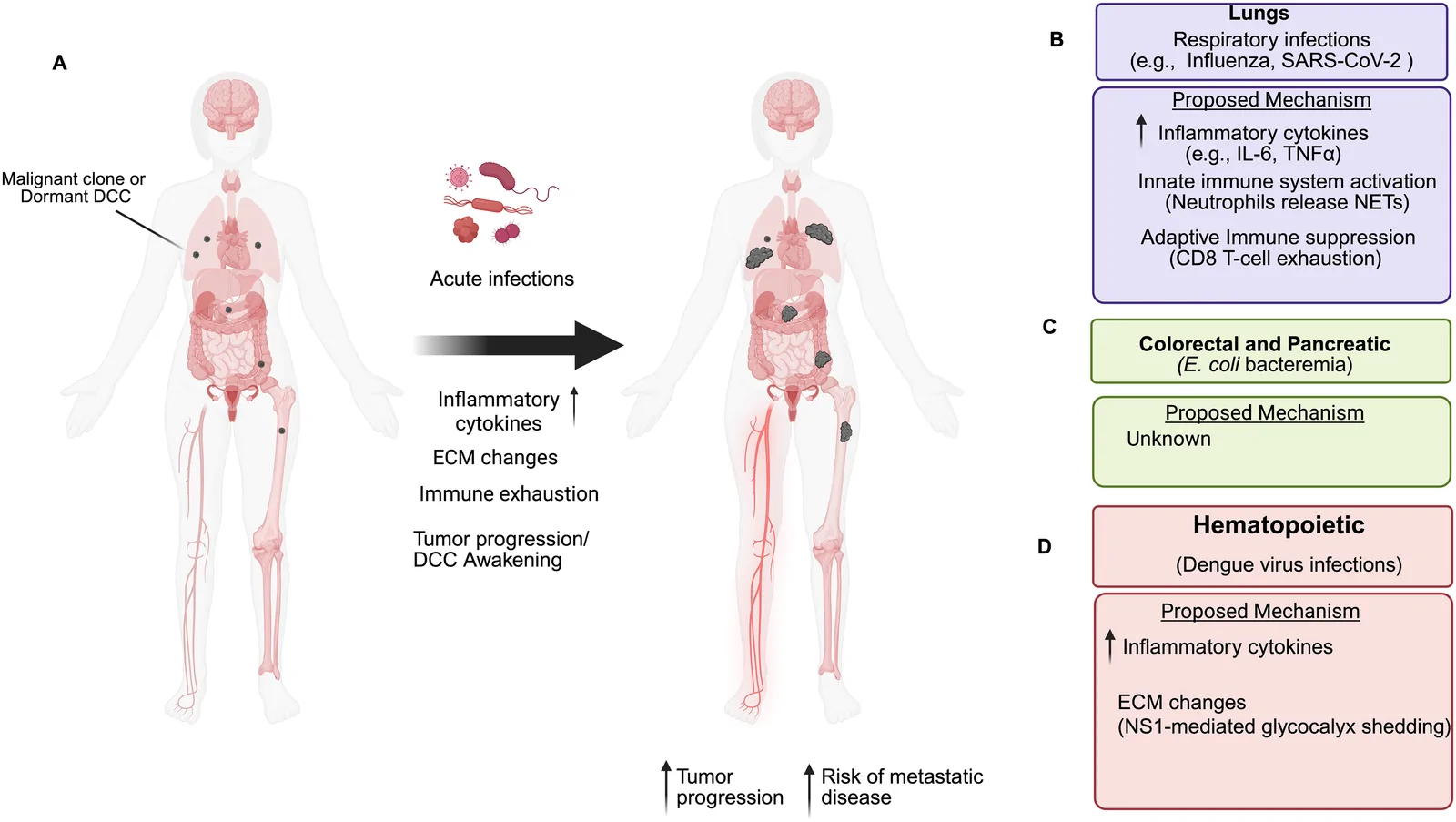
Potential roles for inflammatory cytokines, extracellular matrix changes, and immune exhaustion in promoting tumor outgrowth following acute infections. Respiratory viral infections such as influenza or SARS-CoV-2 can awaken dormant disseminated cancer cells (DCCs) or promote tumor progression in the lungs **(A, B)**. *Escherichia coli* bacteremia has been linked to an increased incidence of colorectal and pancreatic cancers **(C)**, and Dengue virus infections have been linked to an increased incidence of leukemia **(D)**. We note that the mechanisms of enhanced cancer progression can be distinct for these different infections and sites (A–D). Created in BioRender. De dominici, M. (2026) https://BioRender.com/61fg03z.

Epidemiological studies suggest that acute respiratory viral infections in adulthood might lead to an increased risk of developing cancer. A 7-year case-control study of people ≥30 years of age showed increased age-adjusted odds ratios (OR) for infections occurring in the year prior to cancer detection: for influenza 1.29 (95% CI, 1.14–1.46); for gastroenteritis 1.60 (95% CI, 1.41–1.82); and for pneumonia 2.36 (95% CI, 1.79–3.13) [24]. Additional studies of influenza viral infection suggest that the risk of developing lung cancer increases with cumulative exposure to influenza: 1–2 exposures, (1.05 OR, 95% CI 1.00–1.11); 3–4 exposures, (1.12 OR, 95% CI 1.00–1.25); 5+ exposures, (1.25 OR, 95% CI 1.13–1.39) [25]. Analyses of cancer survivors from the UK Biobank (all cancer) and Flatiron Health (breast cancer) databases revealed an increased risk of cancer-related mortality and lung metastasis, respectively, in cancer survivors who were infected with SARS-CoV-2 when compared to those who were uninfected [23] (with analyses for the UK Biobank limited to the period before vaccine rollout). More recently, a retrospective clinical analysis demonstrated that patients hospitalized with COVID-19 exhibited increased lung cancer incidence [26]. While these studies focused on adults, studies of acute viral infections in young people suggest a reduced risk of cancer later in life [27]. This observation suggests a potential priming of the immune system in youth that is beneficial to fighting tumors later in life, whereas infections later in life might lead to immune suppression, resulting in a more pro-tumor response (Fig 1A and 1B).

Looking outside of the lungs, Dengue virus, a non-respiratory arbovirus infecting around 400 million people each year, has been linked to a near doubling of the risk of leukemia in a Taiwanese nationwide cohort, with adjusted hazard ratios of 1.89 (95% CI, 1.13–3.16) overall and 2.43 (1.07–5.52) 3–6 years post-infection [28]. A similar link has been reported for West Nile virus (WNV), with two cases of malignant glial tumors appearing 8 months to 2.5 years after severe neuroinvasive disease [29]. However, unlike the Dengue–leukemia association, the WNV–glioma connection is limited to case reports and needs confirmation in larger studies.

Bacterial systemic infections are also associated with cancer progression across multiple organs. Recent clinical studies have shown that bacterial infections often occur in the months before solid cancer diagnoses [30]. Gram-negative bacteremia (including *Escherichia coli, Klebsiella*, and *Pseudomonas*) can indicate hidden solid tumors such as colorectal, pancreatic, liver, urinary tract, biliary, and lung cancers (Fig 1C) with standardized incidence ratios of 2–4 during the first three months, with a 6-month absolute risk of approximately 1%–2% [31]. And in a Danish population-based cohort, community-acquired *E. coli* bacteremia in adults over 50 years old was linked to an increased risk of colorectal, pancreatic, biliary, lung, and urinary cancers, with a standardized incidence ratio up to 4.5 early on, and higher rates in unexplained cases [30,32]. However, reverse causality should also be considered, as cancer-induced immunosuppression and frailty could enhance the risk of bacteremia. Thus, the cancers may enable bacterial infections, not the other way around.

By contrast, tumor regressions have been reported alongside bacterial infections, syphilis, erysipelas, malaria, and other parasite infections [33]. The therapeutic use of attenuated *Mycobacterium bovis* (Bacillus Calmette–Guérin; BCG) in bladder cancer, now a well-established immunotherapy, traces its roots to these early findings [33,34]. Epidemiological data also suggest an inverse relationship between malaria incidence and cancer mortality, with a 10-fold increase in malaria linked to about 20% fewer cancer deaths [35].

Importantly, epidemiological analyses generally establish associations, not causations, and can be subject to confounding factors and reverse causality. In many studies, key variables (such as vaccination status) are unknown, and while confounding factors are often controlled to the extent possible, biases can persist (such as those related to detection or to associated factors). With this in mind, what lessons can be learned from these studies? Clearly, acute infections can alter cancer progression, but the directionality of these effects can vary depending on the type of infection. Given the substantial mobilization of both innate and adaptive immune cells during these infections, that cancers are impacted is not surprising. The question is, what determines whether the resulting immune response help or hinders the cancer?

## What mechanisms might be involved?

While more studies are clearly needed, emerging evidence from both epidemiology and model systems increasingly supports pro-tumor outcomes from respiratory viral infections in some cases, and anti-tumor effects in others. On the one hand, acute inflammation, such as that induced by respiratory viral infection, is one pathway being explored as a way of awakening dormant DCCs or otherwise altering cancer progression. On the other hand, mobilization of anti-tumor immunity following infection has also been suggested to contribute to reductions in cancer.

### Inflammation following respiratory virus infection

Preclinical mouse models of melanoma have shown opposing effects of influenza infection. Following influenza virus infection, one group found a robust anti-tumor CD8 T cell response that controlled tumor growth [36]. By contrast, another group demonstrated a pro-tumor response that resulted in acceleration of tumor growth and cancer-specific death [37]. Interestingly, both studies showed CD8 T cell recruitment from the tumor to the lung following viral infection, suggesting a common underlying biology and also that differences in crosstalk between the tumor and the immune system could account for the opposing results, perhaps reflecting the tumor model used. The study showing an anti-tumor effect of influenza virus infection used the B16-F1 tumor model, while the study showing pro-tumor effects of influenza virus infection used the B16-F10 model. B16-F10 melanoma cells are known to be highly metastatic to the lung, while B16-F1 melanoma cells are poorly metastatic [38–40]. Thus, the pro-tumor effect of influenza virus infection in the B16-F10 melanoma model could be due to the highly inflammatory lung environment following infection (Fig 1B).

Additional studies have highlighted the context-dependent impact of influenza virus infections on cancer. Patients with lung cancer who experienced hospitalization for influenza virus infection during their treatment course exhibited decreased lung cancer-specific and overall mortality, with a prolonged time to mortality [41]. In line with these results, influenza lung infection (intranasal) or influenza vaccination via intratumoral, but not intramuscular, injection reduced tumor numbers and growth (respectively) in mice transplanted with B16-F10 melanoma [41]. Additional studies showed that prior influenza virus infection in mice led to modestly better control of a subsequently implanted Lewis lung carcinoma [42], apparently due to the generation of antibodies against tumor-associated antigens following the infection. However, these models used implanted cancer cells, with associated neo-antigens. These models rarely produce the long-term presence of dormant DCCs that persist and are associated with immune tolerance [43]. Dormant DCCs are assumed to be immune-privileged [44,45], given that they can persist for up to decades in humans [46–48].

In a mouse breast cancer model, dormant DCCs in the lung awaken and expand following infection with influenza virus or SARS-CoV-2 [23]. This awakening of DCC, expansion, and development of breast cancer metastases in the lung are dependent on interleukin-6 (IL-6) produced in the lung following infection with either influenza virus or SARS-CoV-2 (Fig 1) [23]. The maintenance of expanded DCCs post-infection involves CD4 T-cell-mediated inhibition of CD8 T-cell anti-tumor cytotoxicity. A more recent study showed that influenza virus or SARS-CoV-2 infection promoted *Kras*/Trp53 mutation-driven tumor growth in the lung (Fig 1A), and this growth was mediated by immune suppression [26]. Influenza virus infection also promotes lung tumor growth by exhausting memory CD8 T cells and creating a more immunosuppressive microenvironment (Fig 1B) [37,49].

This trade-off, shaped by evolutionary pressures on host immune cells to prioritize pathogen clearance, equips acute inflammation with a double-edged sword: it effectively combats infection but also risks bystander promotion of tumor growth, metastasis, or even autoimmunity. In cancer models, viral inflammation can paradoxically enhance neoantigen cross-presentation by dendritic cells and boost tumor-specific CD8 T cell expansion [36], yet more often suppresses immunity via myeloid reprogramming, cytokine storms, and exhaustion markers, tipping the balance toward cancer escape and outgrowth. The context dependency (e.g., pathogen type, timing, viral load, pre-existing tumor burden) can help explain why influenza drives melanoma progression in some models [37] but trains alveolar macrophages for anti-tumor protection in others [50].

As indicated above, epidemiology cannot establish causation, and is subject to confounding factors. Experimental models can provide cause-and-effect results but are inherently contrived systems. While we can speculate on the features of different models and population-level results that indicate opposing effects of acute respiratory virus infections on cancer outcomes, the important point is that outcomes can be opposing, even for the same virus. Understanding these immune pathways and their context dependence could enable targeted interventions, such as modulating neoantigen-specific recall, blocking acute inflammatory mediators, or using post-viral adjuvant therapies, to turn tumor promotion into tumor control, transforming acute infections from metastatic threats into opportunities for immunotherapy.

### Inflammation following other types of infections

Similar inflammatory states also arise during other viral infections and systemic bacterial infections. In these settings, NETs, eosinophils, myeloid-derived cytokines such as IL-6 and tumor necrosis factor (TNF), ECM changes, and immune evasion may act together to create a permissive environment for tumor growth and metastasis [51].

In mouse models, the non-respiratory virus lymphocytic choriomeningitis virus (LCMV), which infects the spleen, liver, and kidneys, can engender either acute or chronic infections. Using a mouse lung cancer model and challenge with either acute or chronic LCMV, acute but not chronic infection was able to control tumor growth [52]. However, in the context of melanoma, mice with the highly metastatic B16-F10 tumors showed accelerated cancer-specific death following acute infection with LCMV [37]. As above, exploration of the differences in these models that result in opposite impacts on cancer outcomes following infections with the same virus could provide valuable insights that could lead to interventions that favor the preferred outcome—tumor control.

For systemic infection with Dengue virus, the proposed mechanisms linking infection to cancer progression include NS1-mediated endothelial glycocalyx shedding, changes in myelopoiesis via platelet–monocyte aggregates, and prolonged increases in IL-6 and TNF that remodel bone marrow stroma into a leukemogenic niche (Fig 1D) [28]. While more speculative, acute inflammation triggered by other non-respiratory viruses, such as chikungunya or WNV, might also have the potential to reprogram diverse tissues beyond the lung into permissive metastatic niches, supporting cancer progression in bone marrow, the central nervous system, and other organs. Although the evidence remains limited and, in some cases, preliminary, emerging epidemiological observations have raised the possibility that these infections may be associated with altered cancer risk or patterns of metastasis; systematic surveillance, better diagnostic workups, and careful longitudinal follow-up in patients with cancer who have acute arboviral or neurotropic infections will be essential to quantify their true impact on cancer outcomes.

As for viruses, bacterial infections can promote inflammation and alter adaptive immune responses. Experimentally, acute bacterial lung infection (*E. coli*) or LPS challenge increases pulmonary metastases by 5- to 10-fold in mice for prostate (RM-1), breast (4T1), and melanoma (B16F10) cells [53]. This occurs via extracellular ubiquitin in bronchoalveolar lavage fluid binding to the chemokine receptor CXCR4 on tumor cells; blocking CXCR4 or using antibiotics prevented this effect [53]. In fact, contamination of cigarette smoke with bacterial products such as LPS may contribute to the ability of smoking to awaken dormant DCCs in the lungs [11]. Similarly, pulmonary *Staphylococcus aureus* (Gram-positive bacterium) infection promotes breast cancer lung metastasis through NETs formed via autophagy [54]. In addition, infection with *S. aureus* can accelerate melanoma growth by disrupting anti-tumor CD8 T cell responses [37]. These findings in mouse models reveal how Gram-positive bacteria, such as *S. aureus*, can also drive cancer progression and/or metastases, and correlate with the previously described association of Gram-positive bacterial infection with cancer in humans [55].

By contrast, harkening back to Coley’s toxin and decades of vaccine research, bacteria can function as potent adjuvants to stimulate adaptive immunity. Mechanistically, since the discovery and characterization of innate immune receptor families such as Toll-like receptors (TLRs), many bacterial components (including natural ligands such as flagellin, bacterial DNA, bacterial proteases, and parasite structural components) have been evaluated as immunomodulators and adjuvants in cancer treatment protocols [15,56,57]. Cytokines such as IL-12, interferon-γ, and TNF, which are induced in this context, help eliminate tumors by recruiting infiltrating immune cells, including natural killer cells, macrophages, and dendritic cells, to the tumor site [58].

Protozoans are notable among microorganisms for their substantial clinical and experimental evidence of anti-tumor effects, despite being parasites in humans and animals [59,60]. Studies indicate that they inhibit cancer progression mainly by activating the immune system to reduce tumor growth, angiogenesis, and metastasis in animal models. *Trypanosoma* species (e.g., *T. cruzi*), *Toxoplasma gondii*, and *Plasmodium* species (malaria) show the strongest activity against melanoma, colon adenocarcinoma, and lung carcinoma [59–61]. Generally, parasites like *Plasmodium* seem to trigger strong anti-tumor immunity. While the mechanisms are not clear, it is possible that the multifunctional T cell response (primarily CD8 T cells and CD4 T helper 1 cells) triggered by *Plasmodium* [62] could lead to cross-reactivity with tumor antigens and facilitate the elimination of the tumor cells.

This duality, shaped by evolutionary pressures on host immune cells, enables microbial signals to sometimes yield a bystander protective effect against tumors, while more often inadvertently fostering tumor progression. Understanding this balance will be crucial for designing immunotherapies that harness protective contexts while mitigating pro-metastatic risks. Moreover, as most of the studies of the impacts of non-respiratory viral, bacterial and parasitic infections have been in experimental models, testing these ideas in clinical trials will be critical, with careful consideration of risks and benefits; for example, how can these infections be ‘tuned’ to maximize the promotion of anti-tumor immunity while minimizing the types of inflammation that can promote both tumor growth and host toxicity?

## Can sterile inflammation promote cancer progression?

Non-infectious acute inflammation, caused by diagnostic (biopsies) or therapeutic procedures (chemotherapy, surgery), can mimic infection by creating pro-inflammatory environments that activate dormant DCCs and promote metastatic spread [12,63]. Biopsy-induced injury in patients with breast cancer and in mouse models induce persistent infiltration of macrophages, eosinophils, and neutrophils, along with increased levels of IL-6 and enhanced tumor cell growth at the wound edge [64,65]. This results in a 3- to 5-fold rise in lung micrometastases without affecting the primary tumor size [64]. These biopsy-driven effects are prevented by ibuprofen, IL-6 knockout, dexamethasone, or blockade of the chemokine CCL2, and have been similarly observed in glioblastoma [64,66], where biopsy-induced injury accelerates growth of non-biopsied lesions, underscoring that local injury can systemically reshape tumor behavior across organs. Finally, the systemic response to surgery can also promote the outgrowth of dormant breast cancer cells at distant sites, in part by preventing a T-cell-mediated immune response [67].

Chemotherapy reinforces this sterile inflammatory relay by injuring stromal and endothelial compartments while sparing dormant tumor cells, thereby inducing surges of the cytokines IL-6 and G-CSF, neutrophil influx, and NETosis (the process by which activated neutrophils release decondensed chromatin and granule proteins into the extracellular space to form NETs) that remodel the ECM and mobilize DCCs [11,15,68]. Taxanes such as docetaxel [69] awaken dormant breast cancer cells via MEK/ERK signaling in organoid and murine models, generating more proliferative, invasive, and therapy-resistant clones that can be restrained by MEK inhibition or IL-6/G-CSF blockade [69]. Conventional agents such as cisplatin and cyclophosphamide trigger rapid pulmonary neutrophil recruitment and NET formation, with NET-associated proteases cleaving collagen IV and laminin, exposing pro-proliferative integrin epitopes, and releasing damage-associated molecular patterns [70,71]. TLR9 antagonists or inhibition of protein arginine deiminase 4 can reduce NET formation and DCC reactivation by 70%–80% and limit multi-organ seeding in orthotopic models [11,70,72,73]. Notably, these chemotherapy effects may be at least in part mediated by microbial-induced inflammation, as chemotherapy is known to promote dysbiosis and compromise the integrity of the intestinal barrier [74,75].

Chronic psychological or physiological stress converges on the same myeloid hubs to drive recurrence from dormancy [76]. In lung and ovarian cancer models, β-adrenergic signaling induced neutrophils to release the calcium-binding proteins S100A8/A9, activating myeloperoxidase and promoting the accumulation of oxidized lipids that stimulate fibroblast growth factor signaling in tumor cells, thereby licensing escape from dormancy and early relapse [76]. High S100A8/A9 levels correlated with faster cancer recurrence in patients with non–small cell lung cancer, and pharmacologic blockade of β2-adrenergic receptors or S100A8/A9 prevented stress-induced awakening in mouse models, highlighting stress–neutrophil–lipid circuits as actionable targets to preserve tumor dormancy [76].

While these results need to be further tested through epidemiological and clinical studies, we can appreciate that inflammation, whether from sterile injury or microbial exposure, may be the common factor in tumor promotion from these diverse insults. How can we learn to convert such tumor-promoting inflammation into anti-tumor immune modulation?

## What are the potential clinical implications?

Having established that acute inflammation potentially has a common role in promoting cancer growth and metastasis in various contexts, including biopsies [12,63], trauma, and bacterial and viral infections [17,23,75], the next step will be to investigate targeted interventions to reduce the risk of cancer progression, particularly among cancer survivors [77].

Annual administration of respiratory vaccines reduces the incidence and severity of infections [75], which should, in theory, indirectly limit cancer progression, including through DCC awakening. Neoantigen boosts following vaccination may also prime the regression of minimal residual disease [66]. Epidemiological and clinical studies will be necessary to establish how vaccinations against infections might alter the risk of cancer progression. Although infection prevention through vaccination is important, approved treatments for severe COVID-19 (e.g., to minimize the pathogenic inflammatory response) offer new opportunities to prevent infection-driven metastatic progression [78,79]. Clearly, such interventions would need to be very safe and not increase the severity of the infection.

Considering the role of IL-6 in the awakening of DCCs following infection with influenza virus or SARS-CoV-2 in mouse models [23], blocking IL-6 or IL-6 receptor (IL-6R) with biologic therapies (i.e., blocking antibodies) could be a potential approach to prevent lung metastases triggered by these infections. In line with the pro-inflammatory effect of this cytokine, anti-IL-6R antibodies (e.g., tocilizumab) are US FDA-approved for a wide range of inflammatory diseases [80]. In addition, tocilizumab treatment is commonly used in combination with chimeric antigen receptor T cell therapies for cancer to prevent life-threatening cytokine release syndrome [80–82]. Importantly, in 2022, IL-6R blockade with tocilizumab received emergency (and subsequently full) US FDA approval for hospitalized patients with COVID-19 requiring oxygen supplementation [79]. The REMAP-CAP trial and RECOVERY data showed increased ventilator-free survival by neutralizing IL-6R, with a meta-analysis suggesting that both tocilizumab and sarilumab had similar beneficial effects [79,83]. However, treatment with these biologic therapies is costly and normally requires a hospital visit, complicating their application for cancer survivors to potentially prevent metastatic progression following respiratory infections. Alternative treatments using small-molecule inhibitors of this pathway that can be taken orally could be more feasible.

IL-6 mediates signaling through activation of JAK1 and JAK2, two members of the JAK family of kinases (JAK1, JAK2, JAK3, TYK2), and leads to the activation of the transcription factor STAT3 [78,84,85]. JAKs also mediate signaling triggered by a number of other cytokines (e.g., IL-2, IL-7, IL-8, IL-15). Inhibition of this pathway has been successfully used for reducing inflammation and pathogenesis in many inflammatory diseases, including rheumatoid arthritis and psoriasis [78,86], and several JAK inhibitors are US FDA-approved for the treatment of different autoimmune diseases [85,86]. Similar to tocilizumab, baricitinib, a JAK1/2 inhibitor, was also authorized for use in treating severe COVID-19, based on results from the Adaptive COVID-19 Treatment Trial (ACTT)-2 and RECOVERY trials [87,88]. These trials demonstrated reductions in COVID-19 mortality through cytokine-storm blockade, with IL-6 identified as a key component of COVID-19-associated pathologies. Thus, these inhibitors have already been tested in the context of respiratory virus infections. JAK inhibitors are also being investigated (but not yet approved) as an alternative to biological blockade of IL-6R (tocilizumab) to control cytokine storm caused by cancer immunotherapies [89–92]. Although JAK inhibitors are not approved as monotherapy for cancer treatment, with the exception of some hematological myeloproliferative diseases characterized by JAK-activating mutations [93,94], the JAK/STAT pathway is known to have a role in cancer cell survival [95,96].

Therefore, JAK inhibitors could potentially be a feasible therapeutic strategy to reduce the risk of DCC awakening and cancer progression during viral infections in cancer survivors, through multiple mechanisms (including through tumor intrinsic effects). While additional preclinical studies are needed, a clinical trial could be envisioned in which these drugs are administered at the time the first symptoms of infection are noticed (typically 2–3 days post-infection). Importantly, the duration of treatment could be less than a couple of weeks, by which time the inflammatory response triggered by infection has typically resolved. This short-term treatment could alleviate the potential safety concerns associated with JAK inhibitors, commonly observed in patients with chronic inflammatory diseases for whom treatment is often lifelong. These safety concerns of long-term treatments with some JAK inhibitors (e.g., tofacitinib) have been recognized by the US FDA and include adverse cardiovascular events as well as an increased risk of malignancies [97–99], most likely due to the sustained suppression of the adaptive immune response. These risks can also be dependent on the specificity of the JAK inhibitors. While baricitinib primarily inhibits JAK1 and JAK2, tofacitinib also inhibits JAK3, which is essential for CD4 and CD8 T cell responses [86,100].

Common anti-inflammatory treatments could also have potential to help minimize the risk of developing metastases following infection. Nonsteroidal anti-inflammatory drugs (NSAIDs) can reduce metastatic progression resulting from biopsy or surgery at the primary tumor site in preclinical models, although clinical studies are necessary before extending these findings to infections and people [32,34,55], where the therapeutic strategy involves suppressing pro-metastatic immune cell subsets at specific anatomical sites [101]. For instance, targeting increased neutrophil density and activity, either by dissolving NETs or inhibiting S100A8/A9-mediated accumulation of oxidized lipids, could potentially help to prevent lung tumor relapse [102].

**Fig 2:**
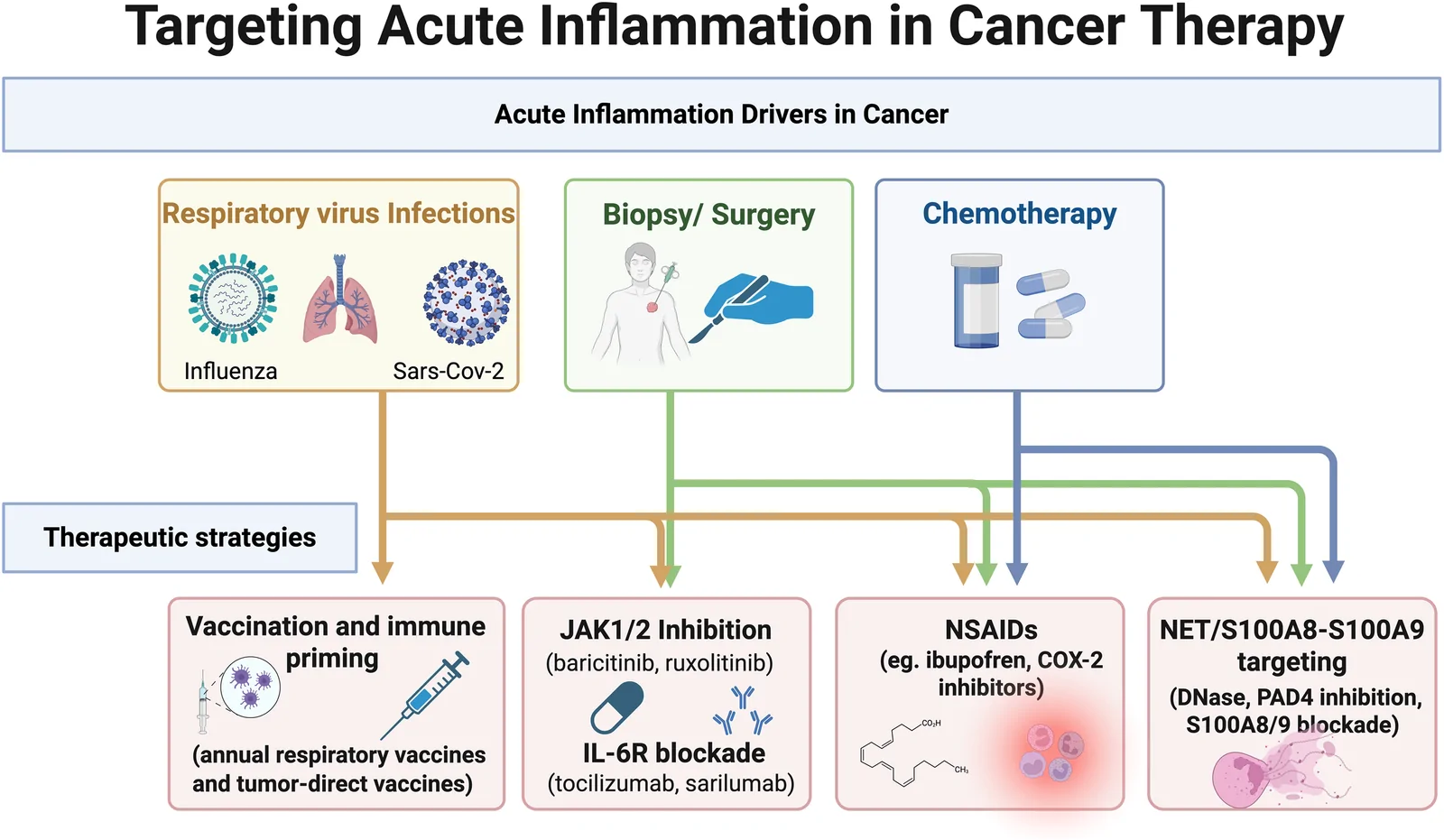
Therapeutic treatments that could potentially resolve acute inflammation that promotes cancer growth. Different triggers (such as respiratory viral infections (influenza and SARS-CoV-2), biopsy/surgery, and chemotherapy) lead to acute inflammation, which promotes tumor growth, disseminated cancer cell (DCC) awakening, and/or metastatic progression. Potential treatments for such acute inflammation include: vaccination and immune priming (annual respiratory vaccines and tumor-associated neoantigen vaccines) to prevent viral triggers; JAK1/2 inhibitors (baricitinib, ruxolitinib) to reduce cytokine signaling; IL-6R blockade (tocilizumab, sarilumab) to counteract IL-6 hypercytokinemia; peri-procedural use of NSAIDs (e.g., ibuprofen or COX-2 inhibitors) to reduce local inflammation during biopsy or surgery; and targeting of neutrophil extracellular traps (NETs) or S100A8/A9 (using DNase, PAD4 inhibitors, S100A8/A9 blockade) to break down NETs and oxidized-lipid niches. These approaches aim to disrupt the inflammation cascade at multiple points by utilizing US FDA-approved drugs with good safety profiles for clinical use. COX-2, cyclooxygenase-2; IL-6, interleukin-6; IL-6R, IL-6 receptor; JAK1/2, Janus kinases 1 and 2; NSAIDs, non-steroidal anti-inflammatory drugs; PAD4, peptidyl arginine deiminase 4; S100A8/A9, S100 calcium binding proteins A8 and A9. Created in BioRender. De dominici, M. (2026) https://BioRender.com/61fg03z.

These potential therapeutic approaches would be feasible and could be tested clinically (Fig 2). Currently, the evidence for the safety and benefits of these treatments remains in the early stages and additional preclinical studies are needed to further support clinical trials. Finally, given that even the same infection (as shown for influenza virus and LCMV) can induce tumor progression in one study and tumor regression in another, a better understanding of the model- and context-dependent differences that underlie such opposing outcomes could lead to additional strategies to convert infection-elicited tumor-promoting inflammation into anti-tumor immunity.

## Conclusions and future directions

How acute infections and the resulting inflammation promotes cancer development and progression, such as by awakening dormant metastasis, is likely a complex interplay of many factors, such as cancer type, severity, duration, cause of the inflammatory stimulus, and type of immune response. Clearly, context matters, and we need to better understand the contexts in which respiratory viral infections can promote cancer progression. In particular, does it need to be a new infection in naïve individuals? The studies in animal models for both influenza virus and SARS-CoV2 infections involved naïve mice [23,26], which does model the early phase of the COVID-19 pandemic but perhaps not the context of repeated infections. More studies in both animal models and data from population studies will be needed to determine how prior infections, the severity of the infection, and other variables such as age, ancestry, and vaccinations alter the impact of infections on cancer pathogenesis.

We also need to expand our understanding of the roles of different immune cell types. In addition to neutrophils and macrophages, recent studies have revealed the presence and role of eosinophils in viral respiratory infections [103,104]. Eosinophils have been found at the edge of the wound in the tumor caused by the biopsy process in patients with breast cancer, and they have also been associated with metastases [65,105]. Future studies should test whether virus-induced eosinophilia contributes to DCC awakening or helps to preserve it.

Understanding why some acute inflammatory stimuli lead to a robust anti-tumor response while others promote primary tumor and metastatic progression will be of critical importance. As we have discussed, elucidation of the differences between experimental and epidemiological studies showing pro-tumor versus anti-tumor impacts of infections could lead to recommendations and even interventions that minimize the former while favoring the latter. Furthermore, recognizing the role of acute inflammation induced by respiratory viruses and other non-viral triggers will be essential for developing protocols for patients currently being treated and for those in tumor remission and recovery to promote a robust anti-tumor response. A significant challenge in translating tumor dormancy treatments into clinical practice is the lack of reliable methods to detect and quantify dormant micrometastases and DCCs in patients [77]. Without precise assessment tools, confirming the complete eradication of these cells remains difficult. As a result, treatment success is typically measured by reductions in relapse rates. While some cancers relapse rapidly, others may remain dormant for decades, which often reduces industry interest and creates financial barriers for academic research. This context underscores the potential impact of interventions that target the immunological components of acute inflammation using currently available drugs. Therefore, protocols for patients who have completed treatment and are at increased risk (such as with detectable cancer burden, even if minimal) should be evaluated and implemented to determine the efficacy of well-established interventions targeting infections and acute inflammation. Importantly, in addition to the key question of how to treat, is the question of whether to treat. Pre-clinical, clinical, and epidemiological studies will be needed to determine the potential benefits and harms of any intervention.

## Abbreviations

ACTT: Adaptive COVID-19 Treatment Trial
DCCs: disseminated cancer cells
ECM: extracellular matrix
IL-6: interleukin-6
LCMV: lymphocytic choriomeningitis virus
LPS: lipopolysaccharide
OR: odds ratios
NETs: neutrophil extracellular traps
NSAIDs: nonsteroidal anti-inflammatory drugs
TLRs: Toll-like receptors
TNF: tumor necrosis factor
WNV: West Nile virus
